# A CpG island hypermethylation profile of primary colorectal carcinomas and colon cancer cell lines

**DOI:** 10.1186/1476-4598-3-28

**Published:** 2004-10-11

**Authors:** Guro E Lind, Lin Thorstensen, Tone Løvig, Gunn I Meling, Richard Hamelin, Torleiv O Rognum, Manel Esteller, Ragnhild A Lothe

**Affiliations:** 1 Department of Genetics, Institute for Cancer Research, The Norwegian Radium Hospital, 0310 Oslo, Norway; 2 Institute of Forensic Medicine, The National Hospital, University of Oslo, Norway; 3 The University Hospital of Akershus, Akershus, Norway; 4 INSERM, Paris, France; 5 Cancer Epigenetics Laboratory, the Spanish National Cancer Centre (CNIO), Madrid, Spain

**Keywords:** *APC*, colon cancer cell lines, colorectal carcinomas, CpG methylation, *E-cadherin*, *hMLH1*, *KRAS*, *MGMT*, MSI, MSS, *p14*, *p16*, *TP53*

## Abstract

**Background:**

Tumor cell lines are commonly used as experimental tools in cancer research, but their relevance for the *in vivo *situation is debated. In a series of 11 microsatellite stable (MSS) and 9 microsatellite unstable (MSI) colon cancer cell lines and primary colon carcinomas (25 MSS and 28 MSI) with known ploidy stem line and *APC*, *KRAS*, and *TP53 *mutation status, we analyzed the promoter methylation of the following genes: *hMLH1*, *MGMT*, *p16*^*INK4a *^(*CDKN2A *α-transcript), *p14*^*ARF *^(*CDKN2A *β-transcript), *APC*, and *E-cadherin (CDH1)*. We compared the DNA methylation profiles of the cell lines with those of the primary tumors. Finally, we examined if the epigenetic changes were associated with known genetic markers and/or clinicopathological variables.

**Results:**

The cell lines and primary tumors generally showed similar overall distribution and frequencies of gene methylation. Among the cell lines, 15%, 50%, 75%, 65%, 20% and 15% showed promoter methylation for *hMLH1*, *MGMT*, *p16*^*INK4a*^, *p14*^*ARF*^, *APC*, and* E-cadherin*, respectively, whereas 21%, 40%, 32%, 38%, 32%, and 40% of the primary tumors were methylated for the same genes. *hMLH1 *and *p14*^*ARF *^were significantly more often methylated in MSI than in MSS primary tumors, whereas the remaining four genes showed similar methylation frequencies in the two groups. Methylation of *p14*^*ARF*^, which indirectly inactivates TP53, was seen more frequently in tumors with normal *TP53 *than in mutated samples, but the difference was not statistically significant. Methylation of *p14*^*ARF *^and *p16*^*INK4a *^was often present in the same primary tumors, but association to diploidy, MSI, right-sided location and female gender was only significant for *p14*^*ARF*^. *E-cadherin *was methylated in 14/34 tumors with altered *APC *further stimulating WNT signaling.

**Conclusions:**

The present study shows that colon cancer cell lines are in general relevant *in vitro *models, comparable with the *in vivo *situation, as the cell lines display many of the same molecular alterations as do the primary carcinomas. The combined pattern of epigenetic and genetic aberrations in the primary carcinomas reveals associations between them as well as to clinicopathological variables, and may aid in the future molecular assisted classification of clinically distinct stages.

## Background

During the last decade, epigenetic changes have been reported in many cancers and they are now recognized to be at least as common as genetic changes [1]. Aberrant methylation of cytosine located within the dinucleotide CpG is by far the best-categorized epigenetic change. The genome of the cancer cell demonstrates global hypomethylation [2,3] as well as regional promoter hypermethylation of several tumor suppressor genes [4]. Hypermethylation of selected CpG sites within CpG islands in the promoter region of genes is associated with loss of gene expression and is observed in both physiological conditions, such as X chromosome inactivation [5], and neoplasia [6]. By inactivating various tumor suppressor genes, this epigenetic modification can affect many important cellular processes, such as the cell cycle (*RB*, *p15*^*INK4b*^, *p16*^*INK4a*^), the *TP53 *pathway (*p14*^*ARF*^), the WNT signaling pathway (*APC*, *E-cadherin*), DNA repair (*MGMT*, *hMLH1*, *BRCA1*), apoptosis (*DAPK*), and the metastasizing process (*E-cadherin*, *TIMP3*) (reviewed in [1,7,8]).

Development of colorectal cancer through various morphological stages has been linked to several genetic and epigenetic changes. The majority of carcinomas have several chromosomal aberrations, a phenotype often referred to as chromosomal instability. Approximately 15% of the tumors are near diploid but exhibit microsatellite instability (MSI), seen as genome-wide short nucleotide insertions and deletions [9]. This phenotype is caused by a defect DNA mismatch repair system [9]. Subgroups of both types of colorectal carcinomas reveal aberrant methylation of tumor suppressor genes leading to lack of expression [10,11].

Human cancer cell lines are important tools in cancer research. Their commercial availability and unrestrained growth make them well suited for *in vitro *studies. Although many of the known genetic aberrations in colon cancer cell lines have been comprehensively described [12], several of these cell lines have not been analyzed for methylation status of pathogenetically important target genes.

The frequencies of both methylation and gene mutation differ among various studies of cell lines and primary tumors. The genome characteristics, profiles of gene mutations, and methylation status are rarely reported in the same samples, let alone in large series. In the present report we address these potentially connected pathogenetic mechanisms by presenting methylation profiles of a set of genes in a series of MSI and microsatellite stable (MSS) colon cancer cell lines and primary colorectal carcinomas. The methylation profiles are compared with various known genetic and clinicopathological features of the same series.

## Results

### Methylation status of target genes in colon cancer cell lines

**Figure 1:**
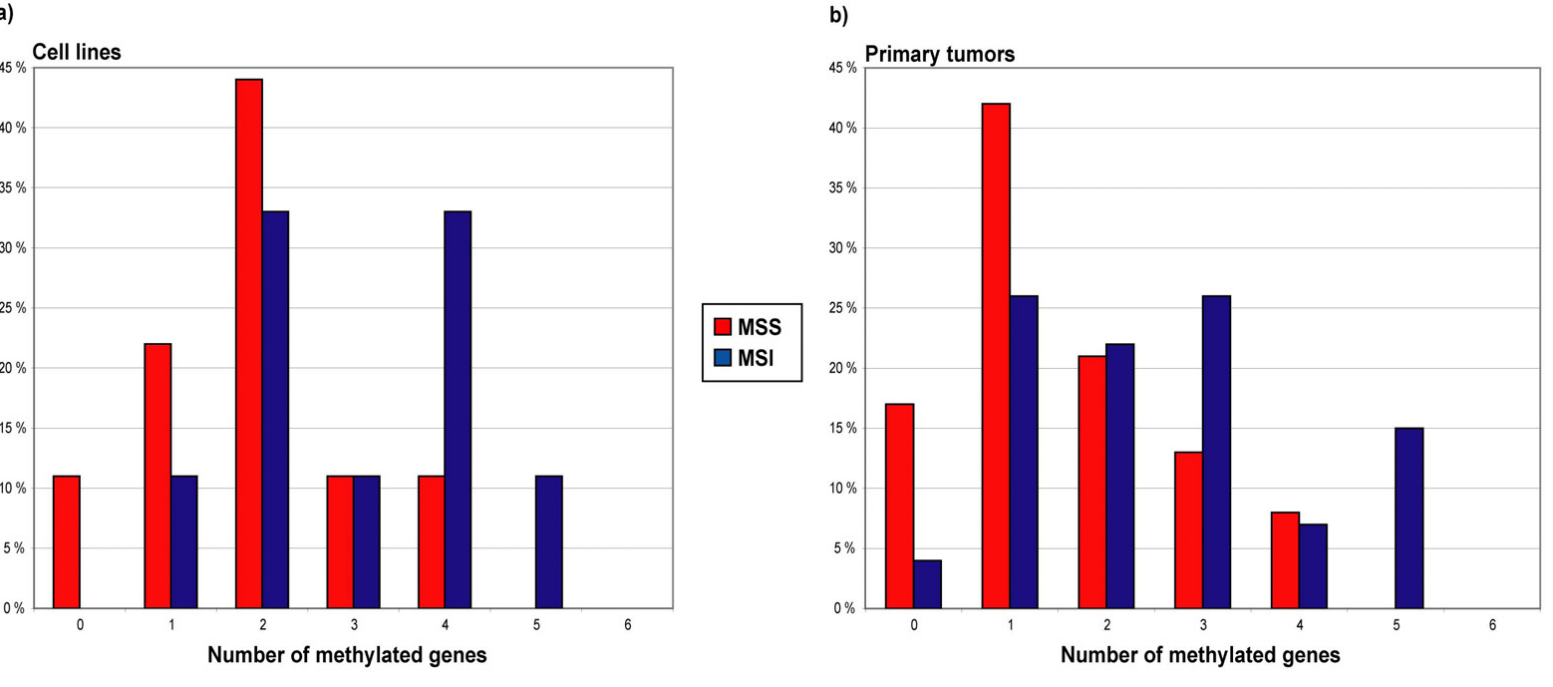
**Distribution of simultaneously methylated promoters in MSS and MSI colon cancer cell lines and colorectal carcinomas. **The two panels illustrate the percentage of MSS and MSI samples displaying methylation of zero to all of the promoters analyzed in the present study in a) cell lines and b) primary colorectal tumors. Abbreviations: *MSS*, microsattelite stable; *MSI*, microsattelite instable.

**Figure 2:**
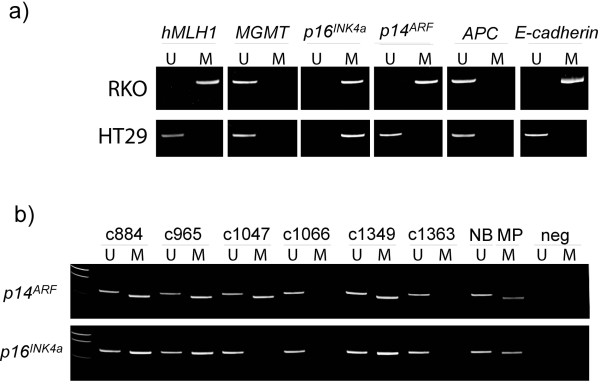
**Promoter hypermethylation in colon cancer cell lines and colorectal primary tumors. **Methylation was evaluated by methylation-specific PCR (MSP). A visible PCR product in *Lanes U *indicates the presence of unmethylated alleles whereas a PCR product in *Lanes M *indicates the presence of methylated alleles. The upper panel (a) illustrates the methylation status of all the loci analyzed in a MSI cell line (RKO) and a MSS cell line (HT29). The lower panel (b) shows the methylation status of representative primary colorectal tumors. Abbreviations: *NB*, normal blood (positive control for unmethylated samples); *MP*, methylated placenta (positive control for methylated samples); *neg*, negative control (containing water as template); *U*, lane for unmethylated MSP product; *M*, lane for methylated MSP product.

The colon cancer cell line methylation-specific PCR (MSP) results are summarized in Table 1 and Figure 1a. Among the MSI cell lines 3/9, 5/9, 7/9, 8/9, 2/9, and 2/9 showed promoter hypermethylation of *hMLH1*, *MGMT*, *p16*^*INK4a*^, *p14*^*ARF*^, *APC*, and *E-cadherin*, respectively, whereas 0/11, 5/11, 8/11, 5/11, 2/11, and 1/11 of the MSS cell lines were hypermethylated for the same genes (Table 2). Hence, the cell lines with MSI generally showed higher methylation frequencies than did the MSS cell lines (Figures 1a, 2a). In most cases, methylation of the target genes was biallelic, but in 10 of the 20 cell lines, monoallelic methylation (detection of both methylated and unmethylated MSP gel bands) was found for one or more of the genes (Table 1). The MSS V9P was the only cell line unmethylated for all six genes analyzed.

**Table 1:** Promoter methylation of colon cancer cell lines. MSI, microsatellite instable; MSS, microsatellite stable; U, unmethylated; M, methylated. The references give results in agreement with our own data except when the reference is underlined. Note that reference 15 does not use the category monoallelic methyaltion, but reports the promoters only as methylated or unmethylated.

	**Cell line**	***hMLH1***	***MGMT***	***p16*^*INK4a*^**	***p14*^*ARF*^**	***APC***	***E-Cadherin***
**MSI**							
	**Co115**	M^12^	M	M^12^	M	U/M	U
	**HCT15**	U^12,13,14,15^	U/M^15,16^	M^12,14,15^	M^14,15,17^	U	U^15^
	**HCT116**	U^12,13,15,18,19,20,21,22^	U/M^15,20^	U/M^12,15,20,21,22,23^	U/M^15,17,21,24^	U/M	U^15^
	**LoVo**	U^12,13,14,15,18,22,26^	U^15,31^	M^12,14,15,22^	M^14,15,24,25^	U	U^15^
	**LS174T**	U^12,13,18,22^	U/M	U^12,22^	U/M	U	U
	**RKO**	M^15,18,19,20,22,26^	U^15,20^	M^15,20,22,27^	M^15,24^	U	M^15^
	**SW48**	M^12,13,14,15,18,20,22,26,28,29^	M^15,20,31^	M^12,14,15,20,22,27,29^	M^14,15,24^	U	U^15^
	**TC7**	U^12^	U	U^12^	U/M	U	U
	**TC71**	U^12^	U	M^12^	U	U	U/M
**MSS**							
	**ALA**	U^12^	U	M^12^	U	M	U
	**Colo320**	U^12,14,18,30^	M	M^12,14,27^	U^14^	U^30^	M
	**EB**	U^12^	M	M^12^	U	U	U
	**FRI**	U^12^	U/M	U^12^	U/M	U	U
	**HT29**	U^12,13,14,15,18,21,22,26,30^	U^15,31,32,33^	M^12,14,15,21,22,27^	U^14,15,21,24^	U^30^	U^15^
	**IS1**	U^12,21^	U	M^12,21^	M^21^	U	U
	**IS2**	U^12^	U	U/M^12^	M	U	U
	**IS3**	U^12^	U	U^12^	M	U	U
	**LS1034**	U^12,13^	U/M	U/M^12^	M	U/M	U
	**SW480**	U^12,14,15,19,21,22,26,30^	U/M^15^	M^12,14,15,21,22,27^	U^14,15,21,24,25^	U^30^	U^15^
	**V9P**	U^12^	U	U^12^	U	U	U

**Table 2:** Methylation frequencies among MSS and MSI colon cancer cell lines and primary colorectal tumors. Abbreviations; MSS, microsatellite stable; MSI, microsatellite instable; CRC, colorectal cancer; U, unmethylated;M, methylated. Note that the calculated methylation frequencies of the MSS cell lines includes results from three cell lines derived from the same patient.

	**MSS**		**MSI**		**Total**	
			
**Gene**	**Cell lines**	**CRCs**	**Cell lines**	**CRCs**	**Cell lines**	**CRCs**
		
*hMLH1*	0/11 (0%)	0/25 (0%)	3/9 (33%)	11/28 (39%)	3/20 (15%)	11/53 (21%)
*MGMT*	5/11 (45%)	10/25 (40%)	5/9 (56%)	11/28 (39%)	10/20 (50%)	21/53 (40%)
*p16*^*INK4a*^	8/11 (73%)	7/25 (28%)	7/9 (78%)	10/28 (36%)	15/20 (75%)	17/53 (32%)
*p14*^*ARF*^	5/11 (45%)	3/24 (12%)	8/9 (89%)	17/28 (61%)	13/20 (65%)	20/52 (38%)
*APC*	2/11 (18%)	7/25 (28%)	2/9 (22%)	10/28 (36%)	4/20 (20%)	17/53 (32%)
*E-cadherin*	1/11 (9%)	10/24 (42%)	2/9 (22%)	11/28 (39%)	3/20 (15%)	21/52 (40%)

### Methylation status of target genes in primary colorectal carcinomas. Comparison with colon cancer cell lines

Methylation status was assessable in more than 99% of the total number of analyses (53 tumors × 6 genes = 318 analyses).

The results of the methylation analyses of 53 primary colorectal carcinomas (25 MSS and 28 MSI) are shown in Table 2 and illustrated in Figures 1b and 2b. All the methylated primary tumors examined showed an unmethylated band in addition to the methylated one, probably due to the presence of normal cells. The methylation frequencies varied from 0% among MSS tumors at the *hMLH1 *promoter to 61% among the MSI tumors for the *p14*^*ARF *^gene (Table 2).

Several of the primary tumor samples displayed widespread CpG island methylation (Figure 1b). Eighteen of 52 tumors (35%) were methylated in 3 or more of the 6 genes analyzed. Only 5/52 (10%) of the tumor samples did not show hypermethylation in any of the genes analyzed. We saw no statistical difference in the number of methylated target genes in colon cancer cell lines versus colorectal primary tumors (Mean Rank 32 for primary tumors versus 38 for cell lines, *P *= 0.231, Mann-Whitney test).

### Methylation profiles compared with genetic characteristics

The methylation status of the primary tumors was compared with genetic characteristics of the same tumors (Table 3). In general, higher frequencies of gene methylation were found among diploid than among aneuploid tumors, reflecting the MSI status, but the differences reached statistical significance only for *p14*^*ARF *^(*P *< 0.001) and *hMLH1 *(*P *= 0.015). Sixteen of 49 primary tumors harbored *TP53 *mutations, and all of the tumors with *TP53 *mutations also harbored unmethylated *hMLH1 *(*P *= 0.009). *p14*^*ARF *^hypermethylation was less common in tumors with mutated *TP53 *than in tumors with wild type *TP53*, although this was not statistically significant (*P *= 0.127). Four tumors displayed a G:C to A:T *TP53 *mutation and three of them simultaneously harbored a methylated *MGMT *gene. Four of 11 tumors with G:C to A:T *KRAS (KRAS2) *mutations were methylated at the *MGMT *promoter. Overall, the presence of *KRAS *mutations was not associated with the methylation status of the genes analyzed. Among the 20 tumors with *p14*^*ARF *^methylation, 10 were also methylated at the adjacent *p16*^*INK4a *^gene (*P *= 0.067). Finally, the *APC *promoter was methylated in 17/53 (32%) tumors, and 8/17 (47%) tumors displayed both *APC *mutation and methylation.

**Table 3:** CpG island methylation of selected genes compared with the patients clinicopathological features and tumor genetics. Abbreviations: Gen. Characteristics, Genetic Characteristics; MSI, microsatellite instability; MSS, microsatellite stable; NS, not significant; Clin. and Path. Features, Clinical and Pathological Features. Comparison of different groups were tested with Fisher exact test or Pearsons χ2 test, *P* values are two sided and are considered statistically significant when *P *≤ 0.05. The table is based on primary tumors (53) and not patients (52) *Statistically significant Pearsons χ2 tests with expected count less than 5.

	*hMLH1*			*MGMT*			*p16*^*INK4a*^			*p14*^*ARF*^			*APC*			*E-cadherin*		
	M		U	M		U	M		U	M		U	M		U	M		U

**Individuals**																		
No	11/53		42/53	21/53		32/53	17/53		36/53	20/52		32/52	17/53		36/53	21/52		31/52
**Gen. Characteristics**																		
**Ploidy**																		
Diploid	10		20	10		20	10		20	18		12	11		19	13		17
Aneuploid	1		22	11		12	7		16	2		20	6		17	8		14
*P *value	0.02			NS			NS			< 0.001			NS			NS		
**MSI-status**																		
MSI	11		17	11		17	10		18	17		11	10		18	11		17
MSS	0		25	10		15	7		18	3		21	7		18	10		14
*P *value	< 0.001			NS			NS			0.001			NS			NS		
***TP53***																		
Wild type	11		22	12		21	11		22	16		16	8		25	13		19
Mutation	0		16	7		9	5		11	4		12	7		9	7		9
*P *value	0.01			NS			NS			NS			NS			NS		
wt+non G-A mutation	11		33	15		29	14		30	18		25	13		31	17		26
G-A mutation	0		4	3		1	1		3	1		3	1		3	2		2
*P *value	NS			NS			NS			NS			NS			NS		
***K-Ras***																		
Wild type	8		19	13		14	9		18	12		15	7		20	9		18
Mutation	1		14	6		9	3		12	2		12	6		9	6		8
*P *value	NS			NS			NS			0.08			NS			NS		
wt+non G-A mutation	8		23	15		16	9		22	13		18	8		23	10		21
G-A mutation	1		10	4		7	3		8	1		9	5		6	5		5
*P *value	NS			NS			NS			NS			NS			NS		
***APC***																		
Wild type	7		19	12		14	10		16	9		17	9		17	12		14
Mutation	3		23	8		18	7		19	10		15	8		18	9		16
*P value*	NS			NS			NS			NS			NS			NS		
**Clin. and Path. Features**																		
**Sex**																		
Male	2		23	9		16	8		17	6		19	10		15	8		17
Female	9		19	12		16	9		19	14		13	7		21	13		14
*P *value	0.04			NS			NS			0.05			NS			NS		
**Age (years)**																		
<68	2		21	10		13	4		19	7		16	8		15	9		14
≥68	9		21	11		19	13		17	13		16	9		21	12		17
*P *value	0.09			NS			0.07			NS			NS			NS		
**Location**																		
Right	10		8	7		11	7		11	12		6	7		11	7		11
Left	1		19	8		12	9		11	5		14	6		14	8		11
Rectum	0		14	6		8	1		13	2		12	4		10	5		9
*P *value	< 0.001*			NS			0.05			0.01			NS			NS		
**Histologic grade**																		
Poorly differentiated	4		8	7		5	6		6	7		4	5		7	4		7
Moderately differentiated	7		30	13		24	11		26	12		25	11		26	14		23
Well differentiated	0		3	1		2	0		3	0		3	1		2	2		1
*P *value	NS			NS			NS			NS			NS			NS		
**Dukes' classification**																		
A	2		2	3		1	1		3	2		2	0		4	1		3
B	5		22	10		17	8		19	9		17	13		14	12		14
C	2		13	4		11	4		11	4		11	3		12	5		10
D	2		5	4		3	4		3	5		2	1		6	3		4
*P *value	NS			NS			NS			NS			0.07			NS		

Among the tumors with widespread methylation (3 or more methylated genes), 13/18 (72%) tumors demonstrated MSI, whereas 5/24 (21%) were MSS (*P *= 0.080). We found no statistically significant associations between tumors with widespread methylation and presence of *TP53*, *KRAS*, or *APC *mutations.

### Methylation profiles and clinicopathological features

The clinicopathological features and methylation status of the primary tumors are summarized in Table 3. We saw more methylation among tumors from females than in those from males for both *hMLH1 *(*P *= 0.043) and *p14*^*ARF *^(*P *= 0.050). Tumors from patients younger than the mean age (68 years) had a lower methylation frequency for *p16*^*INK4a *^than did tumors from older patients, although this was not statistically significant (*P *= 0.074). There was a strong association between methylation and right-sided tumor location as 10/11 (91%) tumors methylated in *hMLH1 *and 12/19 (63%) of the tumors methylated in *p14*^*ARF *^were located in the right side of the colon (*P *< 0.001 and *P *= 0.005, respectively). There was no statistically significant association between methylation and histological grade. Most of the tumors with *APC *methylation (13/17, 76%) belonged to the Dukes' B group, but the differences were not statistically significant (*P *= 0.068).

Tumors with widespread methylation (≥ 3 loci) are associated with right-sided localization; 10/17 (59%), versus 5/17 (29%) left-sided (*P *= 0.035). We saw no statistically significant associations between presence of widespread methylation and the remaining clinicopathological variables included in the present study.

## Discussion

Tumor cell lines are commonly used as experimental tools in cancer research, including studies designed to assess epigenetic changes. But whereas the genetic aberrations of colon cancer cell lines have been comprehensively described [12], the methylation profiles of potential target genes in the same or similar cell lines are often described only sparingly. A literature survey of the 20 colon cancer cell lines and their methylation status analyzed in this study showed that some cell lines and genes had been extensively studied, whereas others were left undescribed (Table 1). For half of the cell lines included in the present study, both methylated and unmethylated alleles have been found for one or more of the genes studied. As non-neoplastic cells are not found in cultured cancer cell lines, this can not be caused by the presence of normal cells, and although several biological and technical explanations may exist, allele specific methylation seems the most likely interpretation [23,34]. In contrast, admixture of normal cells, tumor heterogeneity and/or monoallelic methylation may explain the coexistence of unmethylated and methylated bands in primary tumors.

It has been debated for some time whether cell lines are more frequently methylated than primary tumors [35]. Regarding overall CpG island hypermethylation, cancer cell lines have in general demonstrated an increased frequency of hypermethylation compared with primary tumors [15]. However, only a limited number of the genes analyzed have shown a statistically significant difference in methylation frequency [15]. Among several cancer types examined, colon cancer cell lines have been shown to resemble the most their respective primary tumor in this respect [36]. For the cell lines and primary tumors included in this study, the fraction of MSI and MSS samples was about the same and we saw no statistical difference in the overall number of methylated target genes in colon cancer cell lines versus colorectal primary tumors. Seemingly, large methylation percentage differences for individual genes were seen (Table 2) but they were statistically significant only for *p16*^*INK4a *^methylation, independent of MSI stratification. Comparisons of *in vitro *tumor cells with primary tumors of each subtype (MSS and MSI) have also shown similar frequencies of *TP53*, *KRAS *and *APC *mutations [12] and ploidy stem line [37], which further supports the conclusion that the *in vitro *system is a suitable experimental tool that closely reflect the *in vivo *situation.

Previously reported variations in promoter hypermethylation frequencies of different tumor suppressor genes in colorectal cancer can be explained by various ratios of MSI versus MSS samples in the series analyzed, different methods for analyzing methylation, the inter-individual variation in scoring of methylated samples, incomplete bisulphite modification, tumor heterogeneity, and the fact that different parts of the gene promoter region in question have been analyzed. In the present study, we used primer sets known to only detect methylation in tumor cells, never in normal tissues from the same patients [24,31,38-42]. The promoter hypermethylation in these areas has also shown an impressive correlation with lack of protein expression, confirming that these are essential regions for gene expression [24,31,38-42]. The *hMLH1 *primers we designed amplify a region of the promoter, in which methylation invariably correlates with the lack of *hMLH1 *expression [18,43,44]. Methylation of this region has only been detected in tumor cells and not in normal mucosa [18,43,44].

As expected, the MSI primary tumors showed more methylation overall than did the MSS group. However, this was only significant for the *hMLH1 *and *p14*^*ARF *^genes, whereas the four additional genes analyzed revealed similar methylation frequencies in the MSS and MSI groups. Promoter methylation of the *hMLH1 *gene was, not surprisingly, found only in tumors and cell lines with MSI, not in the MSS samples. The MSS tumors and cell lines per definition contain functional hMLH1 protein, and transcriptional silencing of *hMLH1 *by hypermethylation is known to be the main cause of MSI in sporadic CRC [26,28,45]. Also *p14*^*ARF *^methylation may have a specific role in MSI tumors, since it seems to be most often inactivated in tumors with wild type *TP53 *(see below). However, the relatively high methylation frequencies of the remaining analyzed genes, and also their overall similar frequency in MSI and MSS samples, imply that they are important in colorectal carcinogenesis independently of tumor site and MSI status.

Inactivation of tumor suppressor genes by promoter hypermethylation has been recognized to be at least as common as gene disruption by mutation in tumorigenesis [1]. Indeed, most types of primary tumors harbor several genes inactivated in this way and some genes, like *p16*^*INK4a*^, have been reported to be methylated consistently in most tumor types analyzed [46]. In colorectal carcinomas, the reported *p16*^*INK4a *^methylation frequencies vary from 18% [47] to 50 % [48] with most of the observations centered around 36–40% [11,27,46,49-51], i.e., slightly higher than our result. Both *p16*^*INK4a *^and *p14*^*ARF *^are more commonly methylated in tumors with MSI than in MSS [10,11,51-53], although we found that the methylation frequency of *p14*^*ARF *^is higher than that for *p16*^*INK4a *^in MSI colorectal carcinomas.

The DNA repair protein MGMT is able to remove promutagenic alkyl groups from O^6^-guanine by an irreversible transfer to an internal cysteine residue [54]. Left unrepaired, the alkylated O^6^-guanine has a tendency to base pair with thymine during replication, thereby introducing a G:C to A:T transition mutation in the DNA [55]. Inactivating promoter hypermethylation of the *MGMT *gene has previously been reported to be associated with G:C to A:T mutations in the tumor suppressor gene *TP53 *[56] and the proto-oncogene *KRAS *[57,58]. Our data support this assumption for *TP53 *but seemingly not for *KRAS*, although no certain conclusions can be drawn from the limited number of samples with G:C to A:T mutations.

The p14^ARF ^protein interacts *in vivo *with the MDM2 protein, neutralizing MDM2's inhibition of TP53 [59]. Less hypermethylation of *p14*^*ARF *^in tumors with mutated *TP53 *than in tumors with wild type *TP53 *has been reported previously [24]. Additionally, several reports have described an inverse relationship between MSI and *TP53 *mutation in colorectal carcinomas [60-62]. The frequent methylation we report for the *p14*^*ARF *^gene in MSI tumors with few *TP53 *mutations is in agreement with a recent study [53] and supports the existence of this alternative pathway for TP53 inactivation.

Inactivation of the *APC *gene is frequent in colorectal and other gastrointestinal carcinomas, usually by truncating mutations [63,64]. An alternative mechanism to inactivate the gene in colorectal tumors is by promoter methylation, and we report a frequency of *APC *methylation in the upper range of what has been seen in previous studies [51,65,66]. Somatic mutations in *APC *are common in colorectal cancer [67,68] and, similar to what has been seen by others [12,22,69], almost half of the tumors displaying *APC *mutations in our study were also methylated. We have not looked at allele-specific mutation, but methylation and mutation in the same tumor might reflect one mutated allele and methylation of the other, in accordance with Knudson's two hit hypothesis. This has previously been demonstrated for *APC *in colorectal cancer samples by Esteller *et. al *[65]. APC has a central role in the WNT signaling pathway, which is suggested to play a part in colorectal carcinogenesis by its constitutive activation. Activation of this pathway results in increased transcription levels of genes like *MYC *and *CCND1 (cyclin D1) *further stimulating cell proliferation [63]. Among the 52 successfully analyzed primary tumors in this study, 35 had altered *APC *caused by methylation (n = 17) and/or gene mutation (n = 26). The *E-cadherin *gene was also methylated in 14/34 tumors with altered *APC*, presumably further stimulating WNT signaling [63]. Interestingly, *APC *methylation seemed to be more common in Dukes B stage tumors.

The present study confirms that methylation of *hMLH1 *in sporadic carcinomas is associated with proximal tumor location in the large bowel [14,21,45,70], as above 90% of the primary tumors harboring a methylated *hMLH1 *promoter were taken from the right side of the colon. An association between sporadic proximal colon carcinomas and methylation has also been reported for *p16*^*INK4a *^and *p14*^*ARF *^[14,21,45]. Among our 53 primary tumors, we can only confirm this statistically for *p14*^*ARF*^. However, *p16*^*INK4a *^demonstrated the same tendency. Both *hMLH1 *and *p14*^*ARF *^are strongly associated with MSI and MSI is in turn strongly associated with proximal tumor location [71,72], hence, it is not unexpected that the methylation of both genes is associated with proximal location.

When it comes to gene methylation and its association with other clinicopathological features, contradictory results have been reported. Our observation that methylation of *p14*^*ARF *^does not exclude *p16*^*INK4a *^methylation, is in accordance with previous studies [21,24]. Correlation of *p16*^*INK4a *^or *p14*^*ARF *^methylation with female gender and increased age has been described in some studies [14,47] but not in others [11,21,24]. We found such an association between female gender and methylation of *p14*^*ARF *^and *hMLH1*, but not of *p16*^*INK4a*^. We also found a weak association between *p16*^*INK4a *^methylation and increasing age. This potential age-specific methylation was not confirmed for any of the other genes studied. The gender-associated methylation of *hMLH1 *has previously been described [73,74] and might explain the increased prevalence of colorectal tumors of the MSI type in the female patient group [74].

Like Toyota *et. al *[51], we found no statistically significant associations between tumors with widespread methylation and age, gender, or stage of the colorectal cancer.

## Conclusions

The data presented here demonstrate that multiple genes are methylated in colorectal carcinomas. This underlines the important role epigenetic inactivation of tumor suppressor genes plays during the process of tumor development. Epigenetic changes in colon cancer cell lines are overall comparable with those of primary carcinomas of the large bowel, which make the cell lines relevant models for the *in vivo *situation. The methylation profile of specific genes, in particular *hMLH1 *and *p14*^*ARF*^, has strong associations with genetic and clinicopathological features and might be related to biologically distinct subsets of colorectal tumors.

## Methods

### Patients and cell lines

Fifty-three primary colorectal carcinomas from 52 patients, including 25 MSS tumors and 28 MSI tumors, were submitted to methylation analyses. One of the tumors was from a patient with hereditary non-polyposis colorectal cancer (HNPCC), whereas the rest of the cases were sporadic [71]. The tumors have known DNA ploidy pattern [75], MSI status [76], as well as mutation status for *TP53*, *KRAS *and *APC *[62,64,77]. The genetic and clinicopathological variables are found in Table 3. Twenty colon cancer cell lines, 11 MSS and 9 MSI, were also included in the study. These cell lines have previously been characterized for MSI status [61,78-80], 31 different genetic alterations [12], and total genome profiles by Kleivi *et. al *[37]. The primary tumors included in the present study are from a series of carcinomas evaluated to contain a mean number of 84% tumor cells [81]. The DNA was extracted by standard phenol -chloroform procedure.

### Methylation-specific PCR (MSP)

Promoter methylation was studied in *hMLH1*, *MGMT*, *p16*^*INK4a*^, *p14*^*ARF*^, *APC *and *E-cadherin *by MSP, a method that distinguishes unmethylated from methylated alleles of a given gene [38]. After bisulphite treatment of DNA, which converts unmethylated but not methylated cytosines to uracil, DNA is amplified by PCR using primers specific to methylated and unmethylated sequences.

One or two μg DNA from each sample was modified as described [82]. Previously reported primer sets were used for amplification of the *MGMT *[31,82], *p16*^*INK4a *^[38,82], *p14*^*ARF *^[24], *APC *[39,40] and *E-cadherin *fragments [41] (island 3). The primers for amplifying unmethylated and methylated *hMLH1 *fragments were designed in accordance with *hMLH1 *promoter methylation and gene expression studies [18,44]. All primer sets (see Additional file 1) were purchased from Medprobe AS (Oslo, Norway).

All the PCRs were carried out in a total volume of 25 μl containing 1 × PCR Buffer (15mM MgCl_2 _or no MgCl_2_; QIAGEN Inc., Valencia, CA), 200 μM dNTP (Amersham Pharmacia Biotech Products Inc., Piscataway, NJ), and 0.625 U HotStarTaq DNA Polymerase (QIAGEN). PCR products were loaded onto 7.5% polyacrylamide gels, stained with ethidium bromide, and visualized by UV illumination. An independent second "methylated reaction" of the MSP was performed for all the samples included in the present study. In cases with diverging results from the two rounds of MSP, we did a third independent MSP round.

Human placental DNA (Sigma Chemical Co., St. Louis, MO) treated *in vitro *with *SssI *methyltransferase (New England Biolabs Inc., Beverly, MA) was used as a positive control for MSP of methylated alleles, whereas DNA from normal lymphocytes was used as a control for unmethylated alleles. Water was used as a negative PCR control in both reactions.

### Statistics

All 2 × 2 contingency tables were analyzed using Fisher's exact test. Three × 2 tables were analyzed by the Pearson χ^2 ^test. Two of the statistically significant cross-tables analyzed by the Pearson χ^2 ^had cells with expected count less than 5, with a minimum count of 2.96 (Table 3). The Mann -Whitney test was in addition performed when appropriate. All *P *values are derived from two tailed statistical tests using the SPSS 11.5 software.

## Authors' contributions

GEL cultured and isolated DNA from all cell lines and carried out the MSP analyses of these and of the patient samples. GEL interpreted the results, performed the statistics and drafted the manuscript. LT participated in the study design, scored the MSP results independently of author 1, and contributed to manuscript preparation. TL was responsible for the update of the *APC *mutation status in the cohort. GIM and TOR have collected the series of human primary tumors and provided all clinical and pathological information. RH provided all cell lines and information about them. ME contributed with scientific discussions of the results and participated in the writing of the manuscript. RAL conceived the study, was responsible for its design and coordination, and contributed in the evaluation of the results and in preparation of the manuscript. All authors have read and approved of the final manuscript.

## Supplementary Material

Additional File 1 Additional file 1 lists the MSP primers used in the present study. Click here for file
