# Supplementary material for: A CpG island hypermethylation profile of primary colorectal carcinomas and colon cancer cell lines
Source: Mol Cancer. 2004 Oct 11;3:28. doi: 10.1186/1476-4598-3-28 (PMC526388; doi:10.1186/1476-4598-3-28)
Supplement: Additional File 1 — lists the MSP primers used in the present study. [file 1476-4598-3-28-S1.pdf]

Table 4. PCR primers used for MSP.
Abbreviations: M, methylated-specific primers; U, unmethhylated-specific primers; Frg. Size, fragment size; An. Temp, annealing temperature.
* The annealing temperature is given in degrees celcius.

Primer set	Sense primer	Antisense primer	Frg. size, bp	An. temp*
hMLH1-M	CGG ATA GCG ATT TTT AAC GCG TAA GC	CGT CCC TCC CTA AAA CG	72	53
hMLH1-U	TGG ATA GTG ATT TTT AAT GTG TAA GT	ATC CCT CCC TAA AAC AAC TAC TAC CCA	71	60
MGMT-M	TTT CGA CGT TCG TAG GTT TTC GC	GCA CTC TTC CGA AAA CGA AAC G	81	58
MGMT-U	TTT GTG TTT TGA TGT TTG TAG GTT TTT GT	AAC TCC ACA CTC TTC CAA AAA CAA AAC A	93	58
p16-M	TTA TTA GAG GGT GGG GCG GAT CGC	GAC CCC GAA CCG CGA CCG TAA	150	63
p16-U	TTA TTA GAG GGT GGG GTG GAT TGT	CAA CCC CAA CCA CAA CCA TAA	151	64
p14-M	GTG TTA AAG GGC GGC GTA GC	AAA ACC CTC ACT CGC GAC GA	122	59
p14-U	TTT TTG GTG TTA AAG GGT GGT GTA GT	CAC AAA AAC CCT CAC TCA CAA CAA	132	59
APC-M	TAT TGC GGA GTG CGG GTC	TCG ACG AAC TCC CGA CGA	98	63
APC-U	GTG TTT TAT TGT GGA GTG TGG GTT	CCA ATC AAC AAA CTC CCA ACA A	108	57
E-Cad-M	GGT GAA TTT TTA GTT AAT TAG CGG TAC	CAT AAC TAA CCG AAA ACG CCG	204	55
E-Cad-U	GGT AGG TGA ATT TTT AGT TAA TTA GTG GTA	ACC CAT AAC TAA CCA AAA ACA CCA	211	58
